# Evaluation of the Physicochemical and Antibacterial Properties of Experimental Adhesives Doped with Lithium Niobate

**DOI:** 10.3390/polym12061330

**Published:** 2020-06-11

**Authors:** Laisa Cruzetta, Isadora M. Garcia, Gabriela de Souza Balbinot, Amanda S. Motta, Fabrício M. Collares, Salvatore Sauro, Vicente C. B. Leitune

**Affiliations:** 1 Dental Materials Laboratory, School of Dentistry, Federal University of Rio Grande do Sul, Ramiro Barcelos, 2492, Rio Branco, Porto Alegre RS 90035-003, Brazil; laisacruzetta@gmail.com (L.C.); isadora.mgarcia@hotmail.com (I.M.G.); gabi_balbinot@hotmail.com (G.d.S.B.); fabricio.collares@ufrgs.br (F.M.C.); 2 Department of Microbiology, Institute of Basic Health Sciences, Federal University of Rio Grande do Sul, Sarmento Leite, 500, Centro, Porto Alegre RS 90050-170, Brazil; amanda.motta@ufrgs.br; 3 Departamento de Odontología, Facultad de Ciencias de la Salud, Universidad CEU-Cardenal Herrera, C/Del Pozo (s/n), Alfara del Patriarca, 46115 Valencia, Spain; salvatore.sauro@uchceu.es

**Keywords:** methacrylate-based materials, dental materials, light-curing of dental adhesives, antibacterial agents, lithium niobate

## Abstract

The aim of the present study was to formulate dental adhesives with different concentrations of LiNbO_3_ and to evaluate their physicochemical and antibacterial properties. A dental adhesive was formulated using methacrylate monomers and photoinitiators and used as a control filler-free group. Subsequently, three experimental adhesives doped with LiNbO_3_ at different concentrations (1 wt.%, 2 wt.%, and 5 wt.%) were also formulated. All the experimental adhesives were assessed to evaluate the degree of conversion (DC), softening in solvent, immediate and long-term microtensile bond-strength (μ-TBS), radiopacity, ultimate tensile strength, and antibacterial activity. The incorporation of 1 wt.% of LiNbO_3_ had no negative effect on the DC of the adhesive resin compared to the control group (*p* > 0.05). We observed a decrease in the percentage of softening in solvent in the group LiNbO_3_ at 1 wt.% (*p* < 0.05). The addition of LiNbO_3_ increased the radiopacity at a concentration above 2 wt.%, and there was also an increase in cohesive strength (*p* < 0.05). The immediate μ-TBS increased for LiNbO_3_ at 5 wt.% (*p* < 0.05), and there was no statistical difference for the other groups compared to the control (*p* > 0.05). After six months, the group with 5 wt.% still presented the highest μ-TBS (*p* < 0.05). The adhesives showed no antimicrobial activity (*p* > 0.05). LiNbO_3_ was successfully incorporated in dental adhesives, increasing the radiopacity and their resistance to degradation. Although LiNbO_3_ offered no antibacterial properties, the reliability of LiNbO_3_ incorporation in the adhesive encourages new tests to better investigate the antimicrobial action of LiNbO_3_ through temperature variation.

## 1. Introduction

The formulation of adhesive systems to improve physical, mechanical, and biological properties as a function of time has been the main objective of scientific studies [1,2]. The quality of the dentin adhesion is related to the effectiveness in penetration of the monomers within the collagen interfibrillar spaces in order to restore part of the mechanical properties of the acid-etched dentine and protect such a hybrid layer from hydrolytic and enzymatic degradation [3,4]. Clinically, restorative failures seem to occur more frequently due to the inadequate sealing and degradation of the adhesive interface over time. Consequently, there is a loss of adhesive strength, which reduces the longevity of composite restorations [5,6].

It is well known that the consequences of the degradation process are the formation of gaps at the adhesive interface and a drop in dental adhesion [7]. Therefore, cariogenic microorganisms from the oral cavity can penetrate through such gaps into the resin–dentin interface and cause secondary caries [7]. The addition of inorganic particles to adhesive systems has been used as a strategy to combat this issue and reduce the degradation of the adhesive layer, along with the improvement of the mechanical properties [8,9] and the antibacterial activity of restorative materials [10].

In order to confer some sort of antimicrobial characteristics to restorative materials, several substances have been incorporated within their formulations [10,11]. It is known that materials with microbial activity are able to decrease the degradation process at the interface with the dental substrates over time [12]. For these reasons, new materials that possess not only biocompatible but also smart bioactive properties are required, so that they can actively participate in the battle to control the reoccurrence of dental caries [13,14]. In several studies, inorganic fillers such as hydroxyapatite [13,14], tantalum oxide [15], zirconia [16], zinc oxide [2], and niobium [1] were added in adhesive systems to improve their properties and adhesion to the dental substrate.

Interestingly, niobium is a transition metal with biocompatible [17] and bioactive properties [18]. It can induce mineral deposition on its surface when it is stored in saliva [18]. Due to such properties, different forms of niobium have been used in the biomedical industry, such as for implant covering [19].

**Figure 1:**
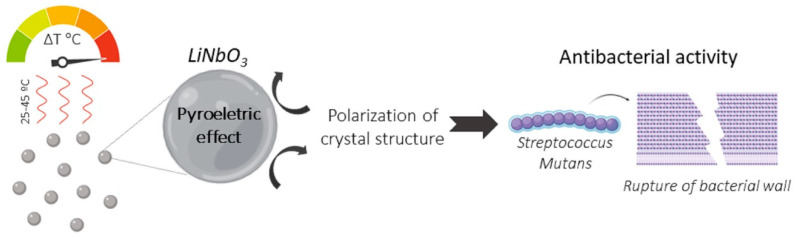
Schematic representation showing the pyroelectric–catalytic effect that occurs at a temperature variation between 25–45 °C, which induces the antimicrobial effect in LiNbO_3_.

Lithium niobate (LiNbO_3_) is an inorganic material that can present a separation of its positive and negative electric charges. Due to its excellent optical, piezoelectric, and pyroelectric properties, LiNbO_3_ has been studied for several applications such as the acoustics surface in televisions, frequency and temperature transformers, infrared detectors, and laser modulators, among others [20]. In addition, LiNbO_3_ has a spontaneously polarized crystal structure, which can be altered by temperature. The change of its polarization after temperature variation may result in the rupture of the cell wall and membranes of bacteria [21]. This phenomenon, known as the pyroelectric–catalytic effect, performs the conversion of thermal energy into chemical energy in the variation between 25–45 °C, which previously showed a high antimicrobial activity (Figure 1) [21]. It is known that restorative materials undergo intraoral thermal cycling from 0 to 67 °C [22]. Thus, the pyroelectric phenomenon of LiNbO_3_ could occur in the oral environment and induce an antibacterial effect, besides improving the bioactivity and the mechanical properties of the adhesives.

The aim of the present study was to formulate dental adhesives with different concentrations (1 wt.%, 2 wt.%, and 5 wt.%) of LiNbO_3_ filler and to evaluate their physicochemical and antibacterial properties. Possible effects were analyzed on the degree of conversion, radiopacity, softening in solvent, ultimate tensile strength, immediate and longitudinal microtensile bond strength, and antibacterial activity regarding the biofilm formation and planktonic cells viability.

## 2. Materials and Methods

### 2.1. Preparation of Experimental Adhesives

**Figure 2:**
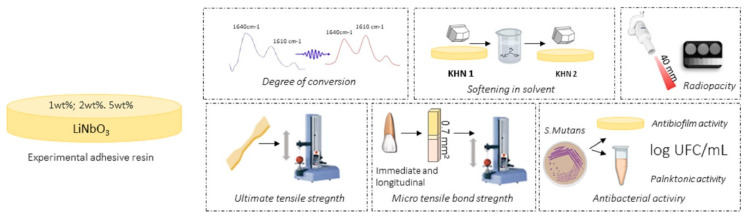
Illustrative image of the adhesives formulated, and methods applied to test the materials: degree of conversion, softening in solvent, radiopacity, ultimate tensile strength, immediate and longitudinal microtensile bond strength, and antibacterial activity.

A dental adhesive was formulated by mixing two methacrylate monomers: bisphenol A glycerolate dimethacrylate (Bis-GMA, Aldrich Chemical Company, St. Louis, Missouri, USA) at 66.66 wt.% and 2-hydroxyethyl methacrylate (HEMA, Aldrich Chemical Company) at 33.33%. Camphorquinone (1 mol%) and ethyl 4-dimethylaminobenzoate (1 mol%) were also used as the photoinitiator and co-initiator, respectively. Butylated hydroxytoluene was used (1 wt.%) as a stabilizer for the inhibition of spontaneous polymerization. These three reagents were also purchased from Aldrich Chemical Company. The adhesive formulated was used as the experimental LiNbO_3_-free control adhesive. LiNbO_3_ was incorporated in experimental adhesive resins at different concentrations (1 wt.%, 2 wt.%, and 5 wt.%). The particles of LiNbO_3_ were milled and sieved (>0.125 mm) and characterized via laser diffraction (CILAS 1180, Orleans, France) for particle size. A light-curing unit (Radii Cal, SDI, Australia) was used for specimens’ photoactivation at 1200 mW/cm^2^. Experimental adhesives were evaluated as illustrated in Figure 2.

### 2.2. Degree of Conversion (DC)

The DC assessment was performed through Fourier Transform Infrared Spectroscopy (FTIR), which was equipped with a spectrometer (Vertex 70, Bruker Optics; Ettlingen, Germany). An attenuated total reflectance device (ATR) was used with the spectrometer. The software was used to adjust the scanning mode parameters, using the Blackman–Harris 3-Term apodization, in a spectrum from 1750 cm^−1^ and 1550 cm^−1^. The resolution used was 8 cm^−1^, and the mirror speed was 2.8 mm/s. Five samples for each group (n = 5) were analyzed. For this intent, each sample was placed on the diamond crystal using a polyvinyl siloxane mold (5 mm in diameter and 1 mm in thickness). The samples were analyzed via FTIR before photoactivation. Another spectrum was acquired immediately after the photoactivation (20 s). The DC was calculated according to an earlier study [23]. For this purpose, the FTIR spectra from the monomer and polymer are used while considering the 1610 cm ^−1^ peak (carbon–carbon double bond in the aromatic chain) as an internal standard, and the peak at 1635 cm^−1^ (carbon–carbon double bond in the aliphatic chain) to calculate the conversion of carbon bonds from monomers to polymers after the photoactivation.

### 2.3. Radiopacity

For the radiopacity test, five specimens, with 6 mm (±0.1 mm) in diameter and 1.0 mm (±0.01 mm) in thickness, were prepared per group (n = 5). One sample per group was placed on the film with an aluminum step-wedge in each digital image acquired. The aluminum step-wedge, as well as the samples, were further analyzed for the pixel density to compare their radiopacity. A digital system was used (VistaScan, Dürr Dental GmbH & Co. KG, Bietigheim-Bissingen, Germany) with a period of exposure of 0.4 s. The distance between the focus and the film containing the samples and the step-wedge was 400 mm. The authors followed a previous study to standardize the parameters and to evaluate the images [1]. The results were expressed in pixel density (mean and standard deviation).

### 2.4. Softening in Solvent (ΔKHN%)

Five samples per group were prepared (n = 5; 1.0 mm thickness × 4.0 mm diameter) to evaluate the initial Knoop hardness (KHN1) with photoactivation for 30 s on each side of the discs. After polishing the samples (silicon carbide sandpapers followed by felt discs with an alumina suspension), they were washed with running water, stored for 24 h, and five indentations (10 g for 10 s) were made on each sample [2]. A microhardness tester (HMV 2; Shimadzu, Tokyo, Japan) was used. The samples were stored in a solution of ethanol (70%) and distilled water (30%) during 2 h. After this period, the samples were tested again, and the final Knoop hardness (KHN2) was acquired. The ΔKHN% was expressed using the difference of the Knoop hardness before and after the immersion in the ethanolic solution.

### 2.5. Ultimate Tensile Strength

The uncured adhesives (n = 12) were placed into a metallic mold and photoactivated for 30 s on each surface. The mold presents an hourglass shape (8 mm long, 2 mm wide, 1 mm thick, 1 mm^2^ cross-sectional area). Each sample had its cross-sectional area measured with a digital caliper before the test. Each sample was attached to a metallic jig with a cyanoacrylate resin, and the jig was placed in a universal testing machine (EZ-LX/EZ-SX Series, SHIMADZU, Kyoto, Japan). The samples are submitted to tensile stress when the upper part of the machine pulls the jig at 1 mm/min. The results in Newtons were divided by the cross-sectional area of each sample. The final results were expressed in megapascals (MPa).

### 2.6. Microtensile Bond Strength to Dentin (μ-TBS)

The bonding performance of the adhesives was analyzed with 96 bovine incisors (n = 12). First, the bovine teeth were cleaned to remove organic tissues (periodontal ligament and pulp). Then, the roots were removed, and the buccal enamel was removed to expose the middle dentin. Each tooth had its buccal surface polished for 30 s with a 600-grit silicon–carbide sandpaper and running water to simulate a smear layer on the dentin. After these steps, the crowns were ready for the adhesion process. Briefly, the dentin was etched (37% phosphoric acid gel for 15 s), water-rinsed (30 s), and dried with absorbent paper. A primer of a commercial three-step etch-and-rinse adhesive system (Scotchbond Multi-Purpose Primer, 3M ESPE; St Paul, MN, USA) was actively applied (20 s). The solvent was evaporated, and the adhesives were applied on the dentin surface. The adhesives were photoactivated for 20 s, and two increments (2 mm each one) of a commercial composite (Filtek Z350 XT Universal Restorative, 3M ESPE, USA) were placed and photoactivated (20 s for each increment).

The restored crowns were immersed in distilled water and kept at 37 °C for 24 h. Then, each crown was fixed in an acrylic device to section it into slices with 0.7 mm of thickness. Water was used to constantly irrigate the crown while cutting it. Each slice was sectioned again to obtain several sticks from each slice (each stick was composed of composite resin, interface area, and dentin). Additional crowns (n = 12) were restored, and the sticks from all those teeth were stored for six months in distilled water at 37 °C. A universal mechanical testing machine (EZ-LX/EZ-SX Series) was used for the microtensile test at 1 mm/min. The values of μ-TBS for the immediate and longitudinal analyses were expressed in MPa.

### 2.7. Evaluation of Antibacterial Activity against Biofilm Formation and Planktonic Bacteria

The antibacterial activity assay was performed according to a previous study [24]. Briefly, the samples of polymerized adhesives (n = 3, 1.0 mm thickness × 5.0 mm diameter) were attached on the lid of a 48-well plate, and this set was sterilized with hydrogen peroxide plasma at 58% during 48 min at 56 °C. On the first day of testing, the wells were filled with 900 μL of brain–heart infusion broth (BHI) containing 1 wt.% of sucrose and 100 μL of a suspension of an overnight broth culture of *Streptococcus mutans* (*S. mutans*, NCTC 10449) at 10^6^ CFU/mL. The set (lid with the samples attached) was placed on the 48-well plate to cover it. Thus, the samples were in contact with BHI and *S. mutans*. Three wells with BHI and *S. mutans* without samples were used as a negative control for the analysis against planktonic bacteria. The set was kept at 37 °C for 24 h. On the second day, the samples were removed from the lid.

To analyze the antibacterial activity against biofilm formation, the samples were immersed in a saline solution in an Eppendorf tube and vortexed. The solution was diluted up to 10^−6^, and two drops (25 μL) from each dilution were placed on the BHI–agar Petri dishes. The dishes were incubated for 48 h at 37 °C for colonies’ growth.

To analyze the antibacterial activity against planktonic bacteria, the BHI in the wells in which the samples were immersed was used. 100 μL of each well was collected and vortexed in 900 μL of saline solution (0.9%), to be diluted up to 10^−6^. Two drops (25 μL) from each dilution were placed on the BHI–agar Petri dishes, and the dished were incubated as described for the antibiofilm activity. After 48 h, the colonies were counted, and the number of colony-forming units (CFUs) per milliliter was expressed for each group.

### 2.8. Statistical Analysis

The data were analyzed using the Shapiro–Wilk test to evaluate their distribution. Then, one-way Analysis of Variance (ANOVA) and Tukey tests compared the adhesives formulated for DC, radiopacity, KHN1 and ΔKHN. A paired t-test was used to compare KHN1 and KHN2 in each group. Two-way ANOVA and Tukey tests compared the μ-TBS data of the different groups at different times (immediate and longitudinal). The tests considered a level of significance of 0.05.

## 3. Results

The laser diffraction analysis revealed an average particle size of 0.55 µm for LiNbO_3_. FTIR spectroscopy was used to analyze the degree of conversion (DC) of the adhesives with and without LiNbO_3_. The peaks at 1610 cm^−1^ and 1635 cm^−1^ was measured in the spectra before and after the photoactivation, and the results were expressed in percentage of C=C conversion into C–C in the monomers’ aliphatic chain (Table 1). The DC values of all groups ranged between 53.55% and 62.51%. The incorporation of 1 wt.% of LiNbO_3_ in the experimental adhesive resin showed no significant difference to the control in the DC (*p* = 0.784).

**Figure 3:**
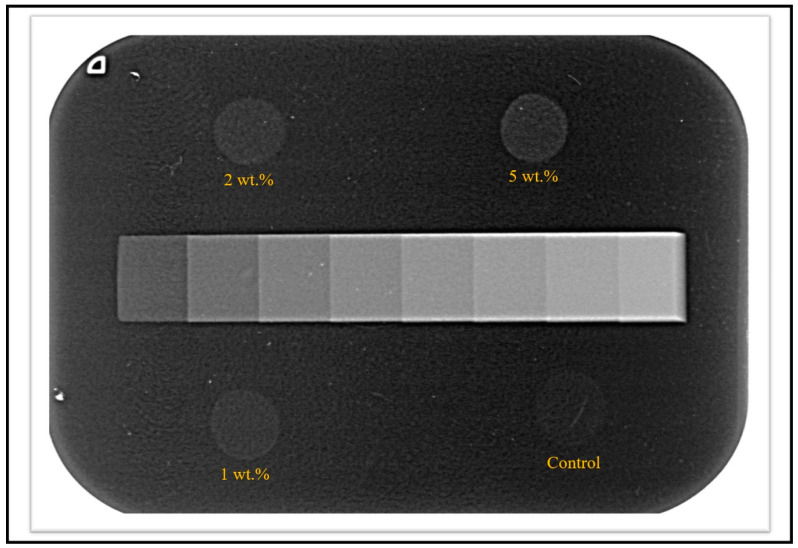
X-ray image obtained in the digital system. One sample per group was exposed to an aluminum step-wedge to compare the radiopacity achieved for each experimental adhesive.

Figure 3 displays an image of the radiographic film with one sample per group acquired during the radiopacity analysis. There was no difference in radiopacity with the addition of up to 2 wt.% of the inorganic filler (*p* > 0.05). However, the 5 wt.% group presented a higher radiopacity than the control group (*p* < 0.001) (Table 1). The results of the ultimate tensile strength test showed that the addition of LiNbO_3_ increased the cohesive strength of the material at a concentration of 2 wt.% compared to the control group (*p* = 0.017). There was no statistical difference between the 1 wt.% and 5 wt.% groups compared to the 0 wt.% LiNbO_3_ group (Table 1).

We observed a higher immediate μ-TBS in the group with 5 wt.% of LiNbO_3_ compared to the control group (*p* = 0.035), but no differences to the 1 wt.% and 2 wt.% groups (*p* = 0.498 and *p* = 0.623, respectively). After six months of storage in distilled water at 37 °C, there was a drop in the μ-TBS values for the specimens in the groups with 1 wt.% and 2 wt.% of LiNbO_3_. There was no significant difference in the control group (*p* = 0.99) and the group with 5 wt.% LiNbO_3_ (*p* = 0.365) when comparing the immediate and the longitudinal μ-TBS. When comparing the values of the longitudinal μ-TBS among the four groups, it was possible to note that the greatest values were obtained for the specimens in the group with 5 wt.% of LiNbO_3_ (Table 2).

There was no difference in the initial hardness (KHN1) among the groups (*p* = 0.092), but all had a reduction in hardness after 2 h of being immersed in ethanol. The degradation rate (ΔKHN%) was lower in the 1 wt.% group (*p* = 0.002), and there was no change in the 2 wt.% and 5 wt.% groups (*p* > 0.05) compared to the control group (Table 3).

The antibacterial activity results are shown in Table 4. No statistical difference was found in the log UFC/mL among groups in the biofilm analysis (*p* = 0.079). In the planktonic analysis, no antibacterial activity (*p* = 0.053) was observed for the LiNbO_3_-containing groups when compared to the adhesives without LiNbO_3_ and the negative control.

## 4. Discussion

Adhesives with inorganic fillers presented improved chemical and mechanical properties [1,2,9]. In addition, their presence within the resin matrix can reduce degradation over time, leading to a longer-lasting material when bonded to the dental substrate [4,25].

The current study demonstrated that the addition of LiNbO_3_ to an experimental adhesive at 1 wt.% decreased the degradation in solvents of the specimens and increased the radiopacity at a concentration of 5 wt.%. Furthermore, the inorganic filler increased the ultimate tensile strength when incorporated at a concentration of 2 wt.%. These characteristics indicate that LiNbO_3_ may be a promising filler for the formulation of innovative resin-based dental materials.

Several studies have shown how the use of inorganic fillers can overcome some degradation problems of the organic matrix [4,26]. The permeation of solvents in the polymer leads to the elution of components and the polymers’ plasticization. This event affects the hardness and the wear resistance of the polymer, and may jeopardize the performance of the restorative treatment over time [5,7,27]. In that context, inorganic particles are less vulnerable to degradation than the polymer, and they may help strengthen the polymeric materials. The addition of LiNbO_3_ at a concentration of 1 wt.% reduced the solubility of the material, making the material less prone to early degradation. SEM images of the adhesives’ surfaces could assist in illustrating this behavior. However, the tests were performed according to previous studies that analyzed the effect of fillers in adhesives. In addition, the analyses were able to demonstrate the behavior of LiNbO_3_ in the resinous matrix.

Softening in solvent is related to the DC of resin-based materials. Polymers with a high conversion of C = C have lower rates of degradation over time [28]. Furthermore, the assessment of the DC of a resin adhesive is essential for obtaining information about the effectiveness of the bond strength of an adhesive for dental substrates [28]. It is expected that inorganic particles, when added to a polymeric matrix, may alter the DC of the material, considering that the refractive index of substances may decrease the availability of light energy within the polymer [29]. Moreover, the addition of inorganic particles may decrease the relative amount of polymer matrix, thus decreasing the polymerization contraction stress and hydrolytic degradation [27,30]. In this study, the addition of 1 wt.% of LiNbO_3_ did not cause any negative effect on the DC values in the experimental adhesive, and the addition of any tested concentration of LiNbO_3_ maintained the DC at a level compatible with commercial adhesives [31]. The quality of the polymer affects the longevity of the restorations [27], and the DC is directly associated with the mechanical properties of polymers [28].

In the antimicrobial test performed in this study, the specimens in contact with the broth containing bacteria were not submitted to a temperature variation. The results showed that specimens containing LiNbO_3_ presented no significant inhibition of bacterial growth (Table 4). This can be explained by the antibacterial action of LiNbO_3_ that occurs due to a pyroelectric–catalytic effect. Indeed, this phenomenon is related to a change in temperature where the filler changes its polarization, generating a charge on its surface that, in contact with the bacteria, may be able to pierce the cell membrane [21]. Therefore, further antimicrobial tests should be performed soon to evaluate how the temperature variation can influence the antibacterial ability of these adhesives.

The addition of LiNbO_3_ at a concentration of 2 wt.% increased the ultimate tensile strength of the material, but at a concentration of 5 wt.% there was no difference compared to the control group. This can be explained because the higher the filler concentration in the resin, the greater the chance of particle agglomerations. Despite the incorporation of inorganic filler to strengthen the organic matrix, in the case of agglomeration it is possible to have zones within the material with altered mechanical properties due to a lack of a uniform distribution of the stress [32]. To overcome this issue, future studies could analyze the possibility of functionalizing the particles of LiNbO_3_ with an organic compound. This method can improve the dispersion of the fillers in polymers [33,34] and keep the particles non-agglomerated [24], which may improve composites’ physical properties.

Commercial adhesives used in current restorative dentistry are not radiopaque, and they may often be confused with lesions underneath the composite restorations, leading to an erroneous diagnosis of secondary caries [35,36]. In this context, radiopaque adhesives would be of great importance in reducing diagnostic errors. According to ISO, radiopaque material has more than 1 mm of aluminum, i.e., materials with values greater than 1 mm are more easily differentiated from dental tissues. The radiopacity values of LiNbO_3_-added adhesives did not reach this value. However, the group with 5 wt.% presented values that were higher than the control group.

The mechanical properties of an adhesive influence the bond strength to the dental substrate [37]. That is, higher mechanical properties promote a more durable bond to the dental substrate. Indeed, it is known that inorganic particles, when added in composite resins, promote an improvement in their mechanical properties [32]. In order to compare the bonding performance of the experimental adhesives, it is necessary to perform the microtensile test that assists in clinical decision making, since there is a correlation between the laboratory tests of bond strength and bond strength in vivo retention in Class V restorations [38].

In the adhesive bond strength test, the addition of LiNbO_3_ increased the immediate bond strength when added at 5 wt.%. Then, the aging of the samples was carried out by storing them in distilled water, which is a well-validated method [5,36]. The results of this study showed that, excluding the experimental adhesive containing 5 wt.% of LiNbO_3_, the bond strength of the other test groups after six months of immersion in distilled water presented no significant changes compared to the control group. In other words, the increased concentration of LiNbO_3_ (5 wt.%) improved the bond strength in comparison to the other groups in the longitudinal analysis. This can be explained by the fact that the addition of inorganic particles may increase the elastic modulus of the polymer, besides improving the stability of the hybrid layer [39].

The addition of LiNbO_3_ increased the radiopacity of the adhesive at a 5 wt.% concentration and did not alter the expected properties of the material at that concentration, therefore being a potential radiopacifying agent. In addition, LiNbO_3_ decreased the softening in solvent when added at 1 wt.% and increased the cohesive strength at 2 wt.%. LiNbO_3_ has been shown to have a high antimicrobial activity when subjected to temperature variation [21], which constantly occurs in the oral environment. Therefore, as a prospective study, a test will be performed to evaluate the antimicrobial activity of adhesives incorporating LiNbO_3_, using these specific parameters.

## 5. Conclusions

In the present research, LiNbO_3_ was added for the first time in a dental material. An experimental adhesive resin was formulated, and different concentrations of LiNbO_3_ were tested as a filler from 1 wt.% to 5 wt.%. The LiNbO_3_ incorporation increased the radiopacity of the adhesive without impairing other physicochemical properties that were tested. The adhesives did not show antibacterial activity, probably due to the lack of temperature changes needed for LiNbO_3_ polarization. However, the reliability of the incorporation of LiNbO_3_ into the adhesive resin showed in this study encourages new tests to better investigate the antimicrobial action of LiNbO_3_ through temperature variation. The possibility of developing a restorative material with a superior radiopacity and improved therapeutic approach via biointeractivity could be further assessed with LiNbO_3_-doped adhesives.

## Figures and Tables

**Table 1:** Results of the degree of conversion (DC), radiopacity, and ultimate tensile strength (UTS) of the experimental adhesives expressed in the mean and standard deviation for each group.

Groups	DC (%)	Radiopacity (Pixel Density)	UTS (MPa)
0% LiNbO_3_	62.61 (±0.40) ^A^	29.45 (±2.56) ^B^	52.81 (±10.11) ^B^
1% LiNbO_3_	61.22 (±2.61) ^AB^	31.37 (±2.70) ^B^	58.90 (±4.14) ^AB^
2% LiNbO_3_	57.99 (±0.45) ^B^	32.27 (±4.92) ^B^	62.04 (±6.90) ^A^
5% LiNbO_3_	53.55 (±3.83) ^C^	38.40 (±3.68) ^A^	57.89 (±4.44) ^AB^

Values followed by different capital letters in the same column indicate statistical difference (*p* < 0.05). Values followed by different lowercase letters on the same line indicate statistical difference (*p* < 0.05).

**Table 2:** The results of the immediate and longitudinal microtensile bond strength (μ-TBS) of the experimental adhesives expressed in the mean and standard deviation for each group.

Groups	Immediate µ-TBS 24 (MPa)	Longitudinal µ-TBS (MPa)
0% LiNbO_3_	31.85 (±13.55) ^Ba^	30.80 (±11.45) ^Ba^
1% LiNbO_3_	38.42 (±9.22) ^ABa^	24.38 (±11.49) ^Bb^
2% LiNbO_3_	38.80 (±12.91) ^ABa^	28.11 (±9.48) ^Bb^
5% LiNbO_3_	45.03 (±8.58) ^Aa^	38.95 (±13.63) ^Aa^

Values followed by different capital letters in the same column indicate statistical difference (*p* < 0.05). Values followed by different lowercase letters on the same line indicate statistical difference (*p* < 0.05).

**Table 3:** The results of the initial Knoop hardness (KHN1), final Knoop hardness (KHN2), and softening in solvent (ΔKHN%) of the experimental adhesives expressed in the mean and standard deviation for each group.

Groups	KHN1	KHN2	ΔKHN%
0% LiNbO_3_	19.64 (±1.05) ^Aa^	11.68 (±1.82) ^b^	40.52 (±8.84) ^A^
1% LiNbO_3_	18.73 (±1.24) ^Aa^	13.68 (±0.84) ^b^	25.59 (±8.14) ^B^
2% LiNbO_3_	19.19 (±0.63) ^Aa^	10.34 (±1.35) ^b^	45.86 (±8.22) ^A^
5% LiNbO_3_	17.48 (±1.94) ^Aa^	8.4 (±2.36) ^b^	51.79 (±10.41) ^A^

Values followed by different capital letters in the same column indicate statistical difference (*p* < 0.05). Values followed by different lowercase letters on the same row indicate statistical difference (*p* < 0.05).

**Table 4:** Antibacterial activity through biofilm and planktonic analyses of the formulated adhesives.

Groups	Biofilm	Planktonic
	Log UFC/mL	Log UFC/mL
0% LiNbO_3_	4.74 (±0.78) ^A^	7.94 (±0.04) ^A^
LiNb_1%_	5.14 (±0.17) ^A^	8.18 (±0.04) ^A^
LiNb_2%_	5.54 (±0.43) ^A^	8.06 (±0.19) ^A^
LiNb_5%_	4.27 (±0.51) ^A^	8.18 (±0.02) ^A^
Negative Control	-	8.00 (±0.03) ^A^

Same capital letters in the same column indicate no statistically significant difference (*p* > 0.05).
